# Pyomyositis in an immunocompetent man

**DOI:** 10.1590/0037-8682-0409-2024

**Published:** 2025-05-09

**Authors:** Elcio Bakowski, Aline Serfaty, Eduardo Alexandrino Medeiros, Gláucia Zanetti, Edson Marchiori

**Affiliations:** 1 Hospital Vivalle, São José dos Campos, SP, Brasil.; 2 Universidade Federal do Rio de Janeiro, Rio de Janeiro, RJ, Brasil.; 3 Medscanlagos Radiologia, Cabo Frio, RJ, Brasil.; 4 Universidade Federal de São Paulo, Escola Paulista de Medicina, São Paulo, SP, Brasil.

**FIGURE 1:**
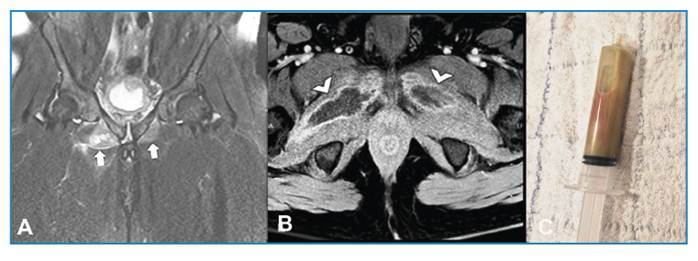
**(A)** Coronal fat-suppressed T2-weighted magnetic resonance imaging (MRI) of the pelvis demonstrating diffuse muscle edema in the bilateral external obturator muscles (arrows). **(B)** Axial fat-suppressed post-contrast T1-weighted MRI showing fluid collections in the muscle bellies, with bilateral peripheral enhancement (arrowheads). **(C)** Aspirated purulent material obtained during CT-guided percutaneous drainage, confirming the diagnosis of pyomyositis caused by oxacillin-sensitive *Staphylococcus*.

A 44-year-old man presented with a history of severe pain in the right groin, particularly during abduction and external rotation of the lower limb, persisting for 2 days. No history of fever or trauma was reported. Blood tests revealed the following: white blood cell count, 18.2×10^3^/μL; C-reactive protein levels, 288 mg/L; and erythrocyte sedimentation rate, 128 mm/h. Magnetic resonance imaging (MRI) of the pelvis revealed fluid collection and diffuse edema in the external obturator muscles (Figures 1A and 1B). Blood culture revealed oxacillin-sensitive *Staphylococcus aureus*. Percutaneous computed tomography-guided drainage of the collections yielded purulent material. The culture results confirmed *S. aureus* infection (Figure 1C), substantiating the diagnosis of pyomyositis. Targeted antibiotic therapy was commenced.

Pyomyositis, a bacterial infection of the skeletal muscle, is characterized by the presence of localized pain, swelling, fever, and occasional abscess formation in advanced stages. Often referred to as “tropical pyomyositis,” pyomyositis is more prevalent in tropical regions; however, it has been observed increasingly in temperate climates, particularly in association with immunocompromised status, diabetes mellitus, recent trauma, and bacterial infections. Imaging plays a crucial role in the diagnosis and management of pyomyositis. MRI, which can reveal diffuse muscle edema with high signal intensity on T2-weighted images and contrast enhancement in the early stages^1^ ^,^ ^2^, is the preferred imaging modality. This report highlights the rarity of pyomyositis in immunocompetent patients without typical risk factors, underscoring the importance of early diagnosis using MRI, timely CT-guided drainage, and targeted antibiotic therapy to achieve favorable outcomes, even in non-tropical regions.
